# Sensory Ataxia Owing to Dysfunction of the Spinocerebellar Tract: A Case Report

**DOI:** 10.7759/cureus.112140

**Published:** 2026-07-06

**Authors:** Ryo Fukata, Atsushi Murata

**Affiliations:** 1 Rehabilitation Medicine, Chiba University Hospital, Chiba City, JPN

**Keywords:** lower extremity dysmetria, romberg sign, sensory ataxia, spinal epidural tumor, spinocerebellar tract

## Abstract

The dorsal spinocerebellar tract (DSCT) conveys unconscious proprioceptive information; however, gait ataxia associated with DSCT dysfunction is clinically uncommon and remains difficult to evaluate using standard neurological examinations. We report the case of a 68-year-old man with thoracic epidural diffuse large B-cell lymphoma causing spinal cord compression at the seventh to eighth thoracic vertebral levels. Following surgical decompression and systemic chemotherapy, muscle strength and conventional conscious proprioceptive findings improved; however, lower-extremity dysmetria and gait ataxia persisted over a 24-month follow-up period. The persistent gait disturbance and positive Romberg sign were compatible with possible DSCT dysfunction, although the evidence was indirect, and subtle posterior column dysfunction or residual myelopathy could not be fully excluded. This case highlights the importance of considering possible DSCT dysfunction in patients presenting with chronic gait ataxia despite apparently normal conventional sensory findings and underscores the value of careful longitudinal assessment for prognosis and rehabilitation planning.

## Introduction

Spinal cord compression secondary to malignant neoplasms is usually accompanied by ataxia and other signs of cerebellar dysfunction in the lower limbs [1]. Nevertheless, cases in which ataxia or dysmetria represent the predominant or sole manifestation of metastatic epidural spinal cord disease are uncommon [1-5]. Sensory ataxia was identified in 1.5% of patients with metastatic epidural spinal cord tumors, whereas severe gait ataxia was the most prominent physical finding in 5% of cases [2]. Therefore, gait ataxia and lower-extremity dysmetria caused by thoracic epidural metastasis are rare and are thought to result from dorsal spinocerebellar tract (DSCT) dysfunction [5]. The DSCT conveys unconscious proprioceptive information from the lower extremities to the cerebellum and contributes to limb coordination and dynamic postural control. Because this pathway is not directly assessed by conventional bedside sensory testing, DSCT dysfunction may be difficult to recognize when vibration and joint position sense appear relatively preserved. Because long-term follow-up data are lacking, the natural history of such ataxia is unclear, and it is generally regarded as irreversible, regardless of treatment timing [5]. Herein, we report a case of thoracic epidural malignant lymphoma in which lower-extremity dysmetria and gait ataxia became clinically identifiable during recovery and were subsequently followed until 24 months after surgery.

## Case presentation

**Figure 1:**
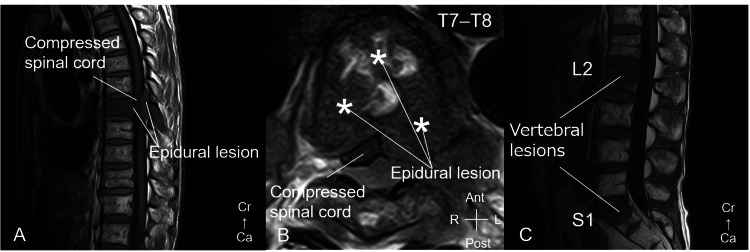
T2-weighted magnetic resonance imaging findings Orientation markers are defined as follows: Cr, cranial; Ca, caudal; Ant, anterior; Post, posterior; R, right; and L, left. A: Sagittal thoracic image showing an epidural lesion at the seventh to eighth thoracic vertebral levels with spinal cord compression. B: Axial thoracic image at the seventh to eighth thoracic vertebral levels showing spinal cord compression by the epidural lesion. Asterisks indicate the epidural lesion. C: Sagittal lumbar image showing additional vertebral lesions at the second lumbar and first sacral vertebral levels.

A 68-year-old man was referred to our hospital after enlarged intra-abdominal lymph nodes were detected on routine ultrasonography. Two weeks later, he developed pain in the thoracic spine at the seventh thoracic vertebral level and in the left flank. Whole-spine magnetic resonance imaging (MRI) revealed osteolytic lesions at the seventh to eighth thoracic, second lumbar, and first sacral vertebral levels, with an epidural mass at the seventh to eighth thoracic vertebral levels compressing the spinal cord (Figure 1). Emergency laminectomy and gross total excision of the epidural tumor were performed. Intraoperative findings confirmed complete decompression of the spinal cord, and pathological examination confirmed a diagnosis of diffuse large B-cell lymphoma.

Postoperative rehabilitation commenced on postoperative day one. Neurological status was evaluated according to the International Standards for Neurological Classification of Spinal Cord Injury. The American Spinal Injury Association Impairment Scale (AIS) was used to classify the severity of spinal cord injury, and the lower-extremity motor score (LEMS) was calculated by manual muscle testing of five key lower-extremity muscle groups on each side, with each muscle graded from 0 to 5 and the total score ranging from 0 to 50 [6]. On postoperative day one, the patient was classified as AIS grade C, indicating motor-incomplete spinal cord injury, with an LEMS of 22. Deep tendon reflexes were brisk in the quadriceps and Achilles tendons; the Babinski sign was absent; and light touch, pinprick, and vibration sensations were intact. Moderate paresthesia was present below the knees, and joint position sense in the big toes was graded 2/5 bilaterally. Standing and ambulation required maximal assistance.

The rehabilitation program was implemented in three phases. During the first four postoperative months at our acute-care hospital, physical therapy was provided five days per week, twice daily, for 40-60 minutes per session. The program included range-of-motion exercises, lower-extremity strengthening, sitting and standing balance training, transfer training, and gait training. Gait training initially consisted of body-weight-supported gait training using bilateral knee-ankle-foot orthoses and progressed to practice with a pickup walker as standing control improved. 

At three months postoperatively, the AIS improved to grade D and the LEMS to 50. Sensory deficits were almost completely resolved; however, ambulation was possible only with a pickup walker. Lower-extremity dysmetria was first identified at this time. The Romberg test was not performed because the patient could not safely maintain unsupported standing with the feet together. No standardized quantitative assessment of ataxia was performed during the first three months.

From four to nine months postoperatively, the patient received intensive inpatient rehabilitation at a rehabilitation hospital seven days per week, three times daily, for 60 minutes per session. The program included repetitive body-weight-supported gait training, gait training with a pickup walker, standing balance training, and activities of daily living practice.

After discharge home at nine months postoperatively, he received home-visit rehabilitation twice weekly for 60 minutes per session. Gait training was progressed from a pickup walker to Lofstrand crutches and then to a single-point cane. Training emphasized safe weight shifting, widening the base of support, and visual monitoring of foot placement. The clinical course and functional outcomes are summarized in Table 1.

**Table 1:** Clinical course and functional outcomes Dysmetria and the Romberg test were not testable on postoperative day 1 due to severe motor impairment and the need for maximal assistance while standing. At 3 months, lower-extremity dysmetria was present, but the Romberg test could not be performed because the patient could not maintain unsupported standing with the feet together. AIS: American Spinal Injury Association Impairment Scale; LEMS: lower-extremity motor score; COP: center of pressure; 10MWT: 10-meter walk test; Dysmetria: +, present; Romberg sign: +, positive; NT: not testable.

Time point	AIS	LEMS	Dysmetria	Romberg sign	Mean COP velocity (cm/s)	10MWT (m/s)	Gait aid
day 1	C	22	NT	NT	NT	NT	Unable to stand
3 months	D	50	+	NT	NT	NT	Pickup walker
12 months	E	50	+	+	NT	0.28	Pickup walker indoors / wheelchair outdoors
24 months	E	50	+	+	4.86	0.32	No assistive device indoors / single-point cane outdoors

At 12 months postoperatively, the AIS was grade E, and the LEMS remained at 50. Sensation, including vibration and joint position sense, was normal; however, lower-extremity dysmetria persisted. Because the patient was able to maintain unsupported standing with the feet together, the Romberg test was performed and was positive. Indoors, the patient ambulated with a pickup walker, and outdoors, he used a wheelchair. He compensated by stiffening the knees, widening the base of support, and visually monitoring foot placement (Video 1). 

**Video 1:** Gait pattern at 12 months postoperatively The patient ambulated indoors with a pickup walker and used a wheelchair for outdoor mobility. His gait was characterized by knee stiffening, a widened base of support, and visual monitoring of foot placement.

At 24 months postoperatively, lower-extremity dysmetria persisted, as demonstrated by the heel-to-shin test (Video 2). The severity of dysmetria was evaluated according to the heel-shin slide item of the Scale for the Assessment and Rating of Ataxia (SARA) [7]. During three cycles, the patient’s heel moved off the shin four or more times on both sides, corresponding to a SARA heel-shin slide score of 3 points bilaterally. The patient also showed an irregular heel trajectory, difficulty maintaining the heel on the opposite shin, and decomposition of movement, consistent with lower-extremity ataxia. He achieved independent indoor ambulation without an assistive device and used a single-point cane for outdoor walking. He no longer relied on a walker or exhibited excessive forward trunk inclination during indoor walking (Video 3). Gait speed in the 10-m walk test was 0.32 m/s. The Romberg test was positive (Video 4).

**Video 2:** Lower-extremity dysmetria demonstrated by the heel-to-shin test at 24 months postoperatively The heel-to-shin test was performed to assess lower-extremity coordination. The patient was instructed to place the heel on the contralateral knee and slide it down along the anterior aspect of the shin toward the ankle. According to the heel-shin slide item of the Scale for the Assessment and Rating of Ataxia, the heel moved off the shin four or more times during three cycles on both sides, corresponding to a score of 3 points bilaterally.

**Video 3:** Independent indoor ambulation without an assistive device at 24 months postoperatively The patient achieved independent indoor ambulation without an assistive device. He used a single-point cane for outdoor walking.

**Video 4:** Positive Romberg sign at 24 months postoperatively The Romberg test was performed to assess postural stability under eyes-open and eyes-closed conditions. However, after eye closure, marked postural sway occurred, and the test was discontinued to prevent falling. This finding indicated impaired postural control when visual compensation was removed.

**Figure 2:**
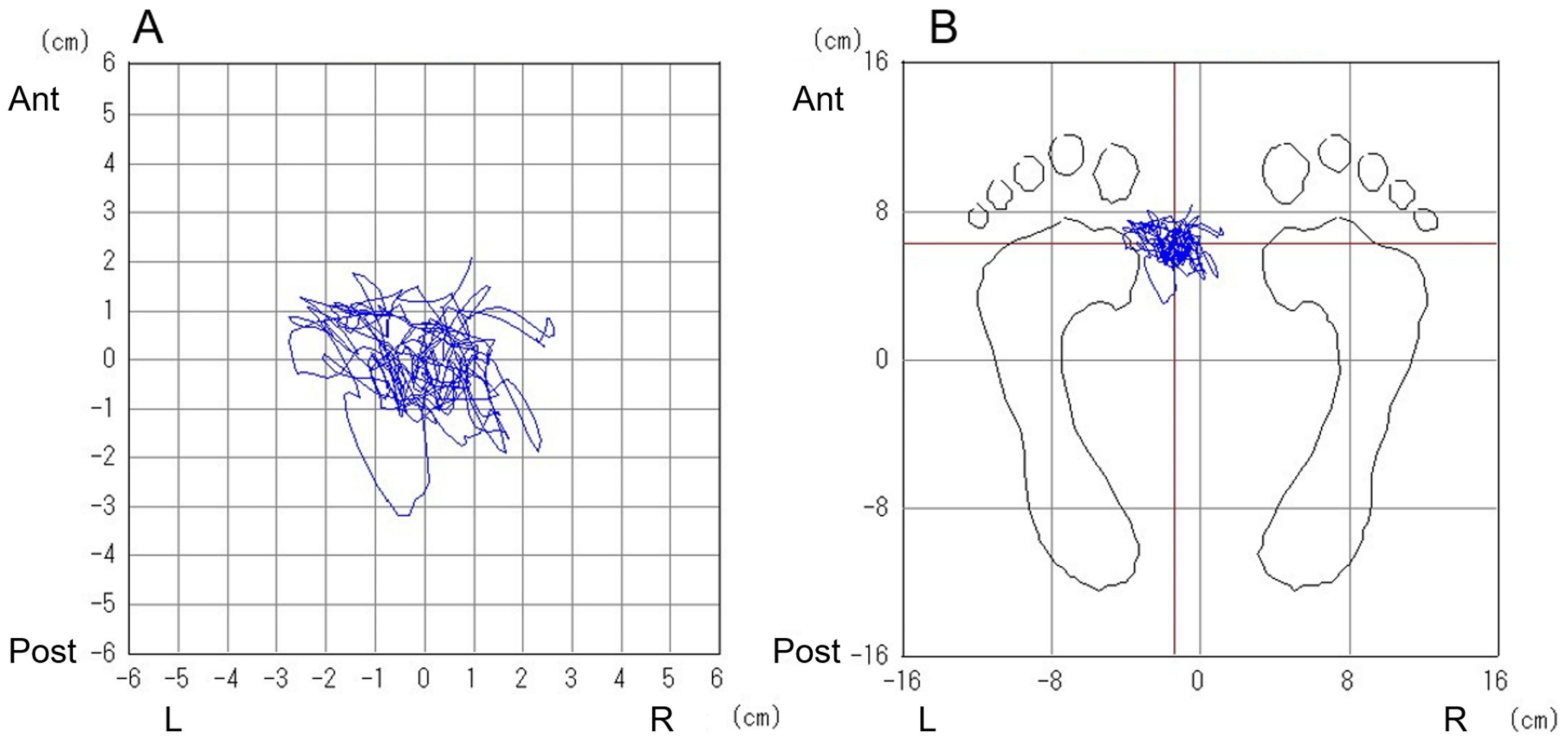
Impaired postural stability assessed using computerized static stabilometry Static stabilometry was performed for 30 seconds under the eyes-open condition using a Gravicorder G-620 system. Orientation markers are defined as follows: Ant, anterior; Post, posterior; R, right; and L, left. A: The mean center-of-pressure velocity was 4.86 cm/s. B: Foot pressure distribution showing anterior displacement of the center of pressure and forefoot-dominant loading.

The patient was able to maintain standing with the eyes open by relying on visual input; however, after eye closure, marked postural sway occurred, and the test had to be discontinued to prevent falling. Therefore, quantitative static stabilometry under the eyes-closed condition could not be performed safely. Static stabilometry was performed using a Gravicorder G-620 system (Anima Inc., Tokyo, Japan) under the eyes-open condition. The patient stood quietly in a barefoot bipedal stance with both upper limbs alongside the trunk for 30 seconds. The mean center-of-pressure velocity was calculated as the total path length of the center-of-pressure trajectory divided by the recording time. The center-of-pressure trajectory demonstrated mediolateral and anteroposterior sway, with a mean center-of-pressure velocity of 4.86 cm/s (Figure 2A). This finding indicated increased postural sway and impaired static postural stability even under the eyes-open condition. Foot pressure distribution showed anterior displacement of the center of pressure and forefoot-dominant loading (Figure 2B). The patient reported the following: “It took a long time, but I gradually noticed an improvement in my walking. The rehabilitation sessions were tough at first, and I often felt frustrated. But little by little, I gained confidence and figured out how to adjust my walking to feel more stable. Now, I’m able to walk outdoors using a single-point cane.”

## Discussion

In the present case, lower-extremity dysmetria was first identified at three months postoperatively. In contrast, the Romberg test could not be safely performed until 12 months postoperatively because unsupported standing with the feet together was not possible earlier. By 12 months postoperatively, the patient had recovered muscle strength and showed no clear deficits in conscious proprioception on conventional neurological examination; however, lower-extremity dysmetria and gait ataxia persisted until the 24-month postoperative follow-up, suggesting possible DSCT dysfunction. Brain MRI revealed no cerebellar pathology. Although joint position sense in the big toes was impaired bilaterally in the acute phase, no clear posterior column signs were detected on conventional follow-up neurological examination.

The DSCT conveys unconscious proprioceptive information from muscle spindles and Golgi tendon organs to the cerebellum and is not directly assessed by conventional bedside sensory testing [8]. Consequently, isolated DSCT dysfunction may be difficult to recognize, particularly during the acute phase of spinal cord injury (SCI). Anatomically, neurons originating from Clarke’s column, which extends from the eighth cervical to the third lumbar spinal cord segments, project ipsilaterally to the cerebellum via the DSCT and convey integrated unconscious proprioceptive information from the lower extremities [8]. 

The AIS is a standard neurological assessment tool for traumatic SCI. However, conscious proprioception is not a mandatory component of the AIS evaluation [6]. Consequently, it has been suggested that even posterior cord syndrome, which involves consciously perceived proprioception, may go undetected in clinical practice [9,10]. Therefore, DSCT injuries, which mediate nonconscious proprioception, may also be overlooked during routine clinical evaluation.

In the present case, persistent lower-extremity dysmetria, gait ataxia, and a positive Romberg sign despite recovery of muscle strength and conventional sensory findings were clinically compatible with possible DSCT dysfunction. Given that the DSCT transmits nonconscious proprioceptive inputs to the cerebellum, its dysfunction may influence both cerebellar and sensory ataxic patterns. However, because neurophysiological testing, follow-up spinal MRI, and tract-specific imaging were not performed, this interpretation remains inferential.

Furthermore, despite marked recovery of motor and conventional sensory functions, gait ataxia persisted for 24 months postoperatively, suggesting that ataxic signs compatible with possible DSCT dysfunction may persist over the long term. Notably, the patient developed compensatory motor strategies, such as knee stiffening, a widened base of support, and visual monitoring of foot placement, enabling independent indoor ambulation without an assistive device and outdoor walking with a single-point cane. These strategies closely resemble those described in previous studies [11]. These findings suggest that even in possible DSCT dysfunction, the nervous system may reweight alternative sensory inputs, such as vision, to maintain postural control.

In this case, the combination of a persistent Romberg sign and lower-extremity dysmetria may serve as clinical clues to possible DSCT dysfunction. Given the difficulty of directly evaluating the DSCT in routine clinical practice, this report highlights how careful clinical observation and specific functional assessments may offer a feasible approach for suspecting DSCT-related pathology. This case underscores the value of detailed analysis of rare presentations for elucidating subtle functional deficits that may be overlooked in larger studies. Thus, this report provides a valuable perspective on the emerging understanding of DSCT-related gait disorders.

This case report has several limitations. First, dysmetria and the Romberg sign could not be assessed in the immediate postoperative period, and no standardized quantitative ataxia assessment was performed during the first three months. Therefore, the exact onset and early progression of ataxic signs could not be determined. In addition, because the patient could not safely maintain standing with eyes closed owing to a positive Romberg sign, stabilometric parameters could not be compared between the eyes-open and eyes-closed conditions. Second, the interpretation of DSCT dysfunction was based on indirect clinical findings because neurophysiological testing, follow-up spinal MRI, and tract-specific imaging were unavailable. Therefore, involvement of other spinal cord pathways, residual myelopathy, or incomplete posterior column recovery could not be fully excluded.

## Conclusions

This case suggests that dorsal spinocerebellar tract dysfunction secondary to thoracic epidural malignant lymphoma may contribute to persistent lower-extremity dysmetria and gait ataxia, even after muscle strength and conscious proprioception have recovered. However, because this interpretation was based on indirect clinical findings, other spinal cord pathways, residual myelopathy, or incomplete posterior column recovery could not be fully excluded. Although these deficits may persist long-term, ambulation can still improve gradually through compensatory motor strategies and rehabilitation. Careful longitudinal assessment of dysmetria and the Romberg sign may help clinicians suspect this condition and guide prognosis and rehabilitation planning.

## References

[1] Gilbert RW, Kim JH, Posner JB (1978). Epidural spinal cord compression from metastatic tumor: diagnosis and treatment. Ann Neurol.

[2] Greenberg HS, Kim JH, Posner JB (1980). Epidural spinal cord compression from metastatic tumor: results with a new treatment protocol. Ann Neurol.

[3] Karp SJ, Ho RT (1986). Gait ataxia as a presenting symptom of malignant epidural spinal cord compression. Postgrad Med J.

[4] García-Moncó JC, Quintana F, Berciano J (1987). Epidural thoracic spinal cord metastasis presenting with cerebellar gait ataxia. Surg Neurol.

[5] Hainline B, Tuszynski MH, Posner JB (1992). Ataxia in epidural spinal cord compression. Neurology.

[6] Rupp R, Biering-Sørensen F, Burns SP (2021). International Standards for Neurological Classification of Spinal Cord Injury: Revised 2019. Top Spinal Cord Inj Rehabil.

[7] Schmitz-Hübsch T, du Montcel ST, Baliko L (2006). Scale for the assessment and rating of ataxia: development of a new clinical scale. Neurology.

[8] Koh M, Markovich B (2026). Neuroanatomy, spinocerebellar dorsal tract. https://www.ncbi.nlm.nih.gov/books/NBK556013/.

[9] Cochrane M, Hess M, Sajkowicz N (2020). Posterior cord syndrome associated with postoperative seroma: the case to perform a complete neurologic exam. J Spinal Cord Med.

[10] Kennamer BT, DelPino BJ, Lettieri SC, Gridley DG, Hollingworth AK, Feiz-Erfan I (2022). Blunt traumatic posterior cord syndrome. Spinal Cord Ser Cases.

[11] Lajoie Y, Teasdale N, Cole JD (1996). Gait of a deafferented subject without large myelinated sensory fibers below the neck. Neurology.

